# Extracellular Vesicle-Based Coatings Enhance Bioactivity of Titanium Implants—SurfEV

**DOI:** 10.3390/nano11061445

**Published:** 2021-05-29

**Authors:** Taisa Nogueira Pansani, Thanh Huyen Phan, Qingyu Lei, Alexey Kondyurin, Bill Kalionis, Wojciech Chrzanowski

**Affiliations:** 1 Department of Dental Materials and Prosthodontics, Araraquara School of Dentistry, São Paulo State University (UNESP), Araraquara 14801-903, Brazil; taisanpansani@gmail.com; 2 Faculty of Medicine and Health, Sydney School of Pharmacy, Sydney Nano Institute, The University of Sydney, Camperdown, NSW 2006, Australia; thanh.phan@sydney.edu.au (T.H.P.); qlei0050@uni.sydney.edu.au (Q.L.); 3 Sydney School of Physics, The University of Sydney, Camperdown, NSW 2006, Australia; alexey.kondyurin@gmail.com; 4 Department of Maternal-Fetal Medicine Pregnancy, Royal Women’s Hospital, Parkville, VIC 3052, Australia; bill.kalionis@thewomens.org.au; 5 Department of Obstetrics and Gynecology, University of Melbourne, Parkville, VIC 3052, Australia

**Keywords:** extracellular vesicle, titanium, implants, osteointegration, bioactivity, osteoblasts

## Abstract

Extracellular vesicles (EVs) are nanoparticles released by cells that contain a multitude of biomolecules, which act synergistically to signal multiple cell types. EVs are ideal candidates for promoting tissue growth and regeneration. The tissue regenerative potential of EVs raises the tantalizing possibility that immobilizing EVs on implant surfaces could potentially generate highly bioactive and cell-instructive surfaces that would enhance implant integration into the body. Such surfaces could address a critical limitation of current implants, which do not promote bone tissue formation or bond bone. Here, we developed bioactive titanium surface coatings (SurfEV) using two types of EVs: secreted by decidual mesenchymal stem cells (DEVs) and isolated from fermented papaya fluid (PEVs). For each EV type, we determined the size, morphology, and molecular composition. High concentrations of DEVs enhanced cell proliferation, wound closure, and migration distance of osteoblasts. In contrast, the cell proliferation and wound closure decreased with increasing concentration of PEVs. DEVs enhanced Ca/P deposition on the titanium surface, which suggests improvement in bone bonding ability of the implant (i.e., osteointegration). EVs also increased production of Ca and P by osteoblasts and promoted the deposition of mineral phase, which suggests EVs play key roles in cell mineralization. We also found that DEVs stimulated the secretion of secondary EVs observed by the presence of protruding structures on the cell membrane. We concluded that, by functionalizing implant surfaces with specialized EVs, we will be able to enhance implant osteointegration by improving hydroxyapatite formation directly at the surface and potentially circumvent aseptic loosening of implants.

## 1. Introduction

Currently, the population aged 60 years or over is ~1 billion. This number is expected to double by 2050. With the increase in aged population, the incidence of osteoporosis, bone fractures, and metabolic diseases has grown concomitantly, increasing the demand for orthopaedic surgeries that require implantable devices. Despite improvements in the functionality of orthopaedic implants, the implantation of metal implants often leads to the formation of avascular fibrotic tissue around the implant. This undesirable process results in insufficient integration of the implant, which diminishes implant functionality, reduces its lifetime, and often requires supplemental surgery [1]. Older patients are more likely to experience some form of health complication that affects bone homeostasis that limits rapid and effective integration of implants into the body [2]. Thus, there is a need for a technology that will accelerate the integration of orthopaedic implants.

To improve osteointegration and the longevity of orthopaedic implants, their surfaces can be modified and functionalised [3]. Currently, there are two major strategies for modifying implant surfaces with the goal of enhancing osteointegration: (a) micro- and nanostructuring of the surface to create ‘cell instructive’ topographies [4,5] and (b) functionalization of surfaces with bioactive molecules that enable cell adhesion and differentiation [4,5,6].

Micro- and nanotopography describe the pattern of roughness of the surface, which affects the cytoskeletal conformation of cells by changing their shape and influencing the adsorption of biological molecules on the surface [7,8]. Topographically structured implants enhance cellular responses such as cell adhesion, proliferation, and differentiation [6,7,8,9,10,11]. The surface functionalization includes chemical treatment and/or the immobilization of inorganic, organic, and biological molecules, such as growth factors, peptides, and proteins [12,13]. Chemically modified implants with additional biomolecules immobilized on their surfaces modulate specific cell signaling pathways and enhance implant integration by stimulating bone formation [14]. While some surface modifications show promise, they are primarily based on functionalization of the surface with one type of molecule (or single topographical feature), which limits potential actions. However, cellular repair requires coordinated activation and inhibition of many different cell types (e.g., stem cells, immune cells, and progenitor cells) to proceed efficiently and successfully. Development of a vehicle containing different biomolecules that could be immobilised on nanostructured implant surfaces would allow construction of specialised cell-instructive surfaces that could significantly enhance implant integration into the body.

Extracellular vesicles (EVs) are nanosize structures secreted by all cells. Their average size is between 20 and 150 nm and their primary function is to mediate intracellular communication and transfer molecular and genetic material between cells [15,16]. EVs contain hundreds of biomolecules that act synergistically to regulate and stimulate different signaling pathways of the recipient cells [16,17]. Thus, EVs play an essential role in tissue regeneration processes [18]. Recently, it was demonstrated that mesenchymal stem cell (MSC) derived EVs contain osteogenesis-related microRNAs (i.e., miR-10b-5p, miR-21, miR-31-3p, miR31-5p, mRi-199a-3p, miR-223-3p) that can induce bone remodeling and mineralization [18,19]. EVs derived from stem cells were found to enhance cell proliferation, angiogenesis, inhibit apoptosis, and decrease inflammation [20]. Furthermore, it was found that decidual mesenchymal stem cells (DMSC) present multipotent differentiation potential, including osteogenic differentiation, and can promote cell migration and bone formation both in vitro and in vivo [21,22]. These characteristics of EVs suggest that, by immobilizing EVs of implant surfaces, we could potentially create bioactive surfaces that stimulate and enhance implant integration.

Moreover, some plants, such as papaya, have healing properties due to the presence of several phytochemicals. Papaya seeds contain phenolic compounds, e.g., β-cryptoxanthin, which have been shown to regulate bone homeostasis and have a stimulatory effect on cell differentiation and mineralization through the enhanced expression of genes involved in bone formation [23,24,25]. Due to these beneficial effects of compounds contained in papaya (i.e., β-cryptoxanthin), we hypothesize that EVs derived from fermented papaya (fermentation is known to result in secretion of large number of EVs) will promote bone cell growth and mineralization.

Therefore, we propose here an innovative approach that harnesses the multipotent nature of EVs to enhance implant integration. Specifically, we developed a two-pronged strategy consisting of: (i) modification/activation of the surface by chemical and plasma treatments and (ii) immobilization of specialized EVs directly at the surface. We hypothesize that the multifaceted actions of EVs would enhance implant integration, their functionality, and longevity.

We foresee that EV-functionalized Ti surfaces—termed SurfEV—will be effective in enhancing the integration of different implantable devices, and their functionality can be tailored by changing the composition of EVs and their concentration. SurfEV could offer an effective solution that will circumvent problems associated with aseptic loosening of implants.

## 2. Materials and Methods

### 2.1. Isolation of Extracellular Vesicles (EVs)

In order to produce EVs, we used: (1) decidual mesenchymal stem cells-DMSC23 cell line (DMSC23-derived EVs-DEVs) [17,22] and (2) papaya fermented fluid (papaya-derived EVs–PEVs; Rocheway, Beeliar, WA, Australia). The human decidual mesenchymal stem/stromal cell line DMSC23 was created by telomerase (HTERT) transduction and retains many of the important properties of the primary decidual mesenchymal stem/stromal cells [22]. Both EV types were isolated using a tangential flow filtration device (TFF) (HansaBioMed Life Sciences Lonza, Tallinn, Estonia).

For DEV isolation, cells were cultured until 80% confluence and maintained in basal medium (Mesencult Basal medium, StemCell Technologies, Vancouver, BC, Canada) containing 0.5% of bovine serum albumin (BSA, Sigma-Aldrich, St. Louis, MO, USA) for 48 h, and then, isolation and concentration steps were performed. For isolation, conditioned medium was transferred into a 50 mL RNase-free centrifuge tube and centrifuged at 500× *g* for 5 min to remove cells and debris. The supernatant was transferred to a new RNase-free centrifuge tube and centrifuged at 2000× *g* for 10 min. The supernatant was syringe-filtered through a 0.45 μm filter (SFCA membrane, Corning^®^, Corning, NY, USA) to TFF device to remove the water and small molecules (<20 nm), and then, the EVs were concentrated in the retainer. For the PEVs, the papaya fermented fluid was submitted to the same cleaning steps, and the supernatant was collected and passed through a 0.45 µm filter (SFCA membrane, Corning^®^, Corning, NY, USA). PEVs were isolated and concentrated using TFF.

As a control for DEVs, we used basal medium (BM; Mesencult Basal Medium, StemCell Technologies, Vancouver, BC, Canada) with 0.5% of BSA (Sigma-Aldrich). The BM was subjected to the same isolation protocol: filtration using 0.45 µm filter (SFCA membrane, Corning^®^, Corning, NY, USA) and isolation using TFF.

### 2.2. Characterization of Size, Size Distribution, Concentration, and Total Nucleic Acid Content of EVs

Following DEVs and PEVs isolation, size, size distribution, concentration, and nucleic acid content were measured using a Flow NanoAnalyzer (NanoFCM Inc., Xiamen, China). As a control for DEVs, the concentration of BM undefined particles isolates was measured using a NanoFCM.

Nucleic acid concertation was measured using SYTO™ RNASelect™ kit (Invitrogen, Eugene, OR, USA) according to manufacture instructions. EVs were stained with 10 μM of green fluorescent cell stain (SYTO RNASelect) at 37 °C for 30 min. The fluorescent events were recorded for 120 s using NanoFCM.

### 2.3. Characterization of the Size and Morphology of Individual EVs

To determine the morphology of individual EVs, we used atomic force microscopy (AFM; MultimodeVII, Bruker, Santa Barbara, CA, USA). EVs were immobilized on a silicon wafer and imaged by AFM operating in tapping mode and using a silicon tip probe (SCOUT 350, NuNano, Bristol, UK): *k* = 42 N/m, 350 kHz. For each EV type, a minimum of 10 images were recorded with at least 10 EVs per image. All images were processed using Mountain8 software (v. 8.0; DigitalSurf, Besançon, France).

### 2.4. Surface Modifications and EV Immobilization—SurfEV

Titanium (Ti) discs were prepared using a three step process comprising: (i) polishing, (ii) alkaline treatment, and (iii) plasma activation. In brief, machined titanium grade 4 Ti discs with 8 mm diameter and 2 mm thickness were polished using SiC paper (Buehler, Germany) with grid size: #600, #800, and #1200. After polishing, samples were washed sequentially in acetone (30 min), propanol (30 min), and then ultrapure water (30 min) using an ultrasonic cleaner and then dried in an oven at 40 °C [26]. Following cleaning and drying, Ti samples were treated in 5 M NaOH solution (NaOH, Kanto Chemical Co., Inc., Tokyo, Japan) at 60 °C under agitation (120 cycles per minute) for 24 h. After NaOH treatment, samples were sonicated in ultrapure water for 10 min to produce a layer of nanostructured sodium hydrogen titanate, approximately 1 μm thick [26,27]. Following this treatment, Ti discs were plasma cleaned using RF plasma (Plasma Cleaner PC-150, Harrick Plasma, Ithaca, NY USA) for 5 min. Nanostructuring and plasma treatment (surface activation) were done to facilitate the attachment of EVs to the surface.

To immobilize EVs on the surfaces, 20 μL of EV solution at a concentration of 100 EVs per cell, 1000 EVs per cell, and 10,000 EVs per cell were applied to each sample and maintained in a desiccator to dry and then used for further experiments.

#### 2.4.1. The Assessment of the Presence of EVs on Ti Surfaces

To determine the presence of EVs on the Ti surface, we used infrared spectroscopy with a Fourier-transform infrared spectrometer operating in reflection mode (FTIR, Excalibur FTS 3000MX, Digilab, Hopkinton, MA, USA). FTIR spectra were recorded by averaging 2000 scans in the range of 3800–400 cm^−1^ with a spectral resolution of 4 cm^–1^. FTIR spectra were recorded for two control samples without EVs (Ti and Ti NaOH + plasma activation) and two experimental samples coated with 20 μL of solution containing 2.5 × 10^6^ DEVs and PEVs, simulating the concentration of 100 EVs/cell on each surface (Table 1). The established density of cells used to analyze proliferation and morphology of cells on Ti surfaces coated with EVs was 2.5 × 10^4^ cells/surface. In order to measure the strength of the EV bond to the surface, all samples were washed with 5 mL of detergent (2% of Sodium Dodecyl Sulphate, SDS, Sigma-Aldrich, St. Louis, MO, USA) solution in deionized water at 25 °C for 2 h with gentle shaking. After an SDS wash, each sample was rinsed three times in deionized water and dried overnight in a Petri dish. FTIR spectra for SDS-washed samples were recorded as described above.

If EVs were bound to the surface only through weak electrostatic or Van der Waals forces, the detergent wash would remove them. However, if covalent bonds were established, EVs would remain on the surface.

In all measurements infrared background spectra were recorded using aluminum foil. To remove the Ti background and obtain information from EVs alone, the FTIR spectrum was recorded for pure Ti (Ti cleaned), and this background value was subtracted from all groups.

#### 2.4.2. The Assessment Calcium (Ca) and Phosphate (P) Deposition on Surfaces—Surface Bioactivity

To investigate the ability of SurfEV to promote the deposition of Ca and P and, potentially, the formation of hydroxyapatite on Ti surface as an indication of their bioactivity or bone bonding ability [26,27,28], we used a protocol established by Bohner et al. [28]. Briefly, the simulated body fluid (SBF) was prepared by dissolving reagent grade NaCl, NaHCO_3_, Na_2_HPO_4_·2H_2_O, CaCl_2_, and HCl (Sigma-Aldrich, St. Louis, MO, USA) in ultrapure water and buffered at pH  =  7.4 [28]. Samples were soaked in tubes containing 5 mL of SBF and incubated and maintained in static conditions at 37 °C and 5% of CO_2_, as recommended by International Organization for Standardization-ISO 23317 [29]. After 3 days in contact with SBF, the deposition of Ca and P on the surface was measured at five areas using energy dispersive X-ray (EDX).

### 2.5. Determination of the Effect of EVs on Cell Responses and Function

To determine the effect of EV concentration on the proliferation, wound closure, and migration of osteoblasts, three different concentrations of EVs (both DEVs and PEVs) were tested: 100 EVs per cell, 1000 EVs per cell, and 10,000 EVs per cell. As a control for DEVs, BM isolates were applied on cells at same tested concentrations (100, 1000, and 10,000 isolates per cell).

Prior to cell seeding, 96-well plates (Corning^®^ Costar, Corning, NY, USA) were treated with RF plasma (Plasma Cleaner PC-150, Harrick Plasma, Ithaca, NY, USA) for 5 min to facilitate effective EV immobilization. After plasma treatment, 5 μL of EVs at each concentration were applied to wells and maintained in a laminar flow hood for 15 min to dry the EV solutions and immobilize them on the surface.

After drying, human osteoblast-like cells (MG63 cell line, [30]) were cultured in a 96-well plate (Corning^®^ Costar, Corning, NY, USA) containing three different concentrations of EVs (100 EVs per cell, 1000 EVs per cell, and 10,000 EVs per cell) immobilized on the bottom of the well plate. Cells were maintained using DMEM culture medium (DMEM—Dulbecco’s Modified Eagle’s Medium, Sigma-Aldrich, St. Louis, MO, USA) containing 10% of fetal bovine serum (FBS, Bovogen Biologicals, Keilor East, VIC, Australia) and 1% of antibiotics (Pen/Strep) (100 units penicillin and 0.1 mg/mL streptomycin, Sigma-Aldrich, St. Louis, MO, USA). For the wound healing assay, 1.7 × 10^4^ cells were cultured per well. For cell migration, cell proliferation, and cell morphology assays 10^3^ cells per well were used.

#### 2.5.1. The Analysis of Ability of EVs to Accelerate Wound Closure

The effect of different concentrations of DEV and PEV on cell migration (wound closure) was determined using a wound healing assay. MG63 cells were seeded in a 96 image-lock well plate (ESSEN Bioscience, Ann Arbor, MI, USA) containing immobilized EVs. After 24 h, a scratch was created in the middle of each well using a wound maker device (ESSEN Bioscience, Ann Arbor, MI, USA). The wells were washed with PBS 1 × RNAse free (Sigma-Aldrich, St. Louis, MO, USA) and replenished with fresh FBS-free medium and then incubated in an IncuCyte (Bioscience, Ann Arbor, MI, USA) for 32 h. Images were obtained every 2 h with a 10× magnification, and changes to the wound width (%) and migration of the front edge of cells were analyzed using IncuCyte ZOOM software (v. 2020C, IncuCyte S3 Base Analysis Software (Essen BioScience, Ann Arbor, MI, USA)).

#### 2.5.2. The Analysis of Effects of EVs on Cell Proliferation

Cell proliferation was measured using the Cell Counting Kit 8 (CCK-8, Donjindo, Japan) assay. This method is based on measures of the dehydrogenase activity with NADH in live cells. MG63 cells were seeded in a 96-well plate (Corning^®^ Costar, Corning, NY, USA) containing immobilized EVs. After 24 h, the CCK-8 solution (10% in serum-free culture medium-DMEM) was added to the cells and incubated at 37 °C for 3 h. The absorbance of the resulting solution was determined by a spectrophotometer at 450 nm, and the proliferation rate calculated according to the mean value of viability obtained for the control group.

#### 2.5.3. The Analysis of Effects of EVs on Individual Cell Migration

Individual cell migration was evaluated measuring the total distance traveled by a single cell during 24 h. MG63 cells were seeded in a 96-well plate at a density of 10^3^ cells per well. The 96-well-plates were prepared as described in #2.5.1 and then incubated in the IncuCyte (Bioscience, Ann Arbor, MI, USA). Images were obtained every 2 h during 24 h. To measure individual cell migration, we used in-house developed software—CellTrack (v. 1.0, 2020, Sydney, NSW, Australia) [31].

#### 2.5.4. The Analysis of Effects of EVs on Cell Morphology

To demonstrate the effects of EVs on cell morphology, cells were imaged using 3D holotomography (NanoLive 3D Cell Explorer Microscopy, Tolochenaz, Switzerland). MG63 cells were seeded in glass bottom Petri dishes (ibidi GmbH, Munich, Germany) and incubated for 24 h. After 24 h, cell culture medium was replaced with new CO_2_ Independent Medium (Gibco, Grand Island, NE, USA) containing EVs at different concentrations (100 EVs per cell, 1000 EVs per cell, and 10,000 EVs per cell). After 1 h incubation with EVs containing medium, images were obtained with a 60× magnification objective lens and processed using STEVE software (v. 1.6.3496, 2020, NanoLive, Tolochenaz, Switzerland).

### 2.6. Elemental Analysis of Cells Using Inductively Coupled Plasma Mass Spectroscopy—ICP-MS

To reveal the effect of DEVs and PEVs on the elemental composition of cells and their potential to promote the mineralization of cells, MG63 cells were cultured in DMEM culture medium supplemented with DEVs and PEVs at a working concentration of 100 EVs/cell. We selected this concentration based on the effect of EVs on osteoblast responses. When osteoblasts were treated with different concentrations of PEVs and DEVs, the 100 EVs/cell concentration did not cause intense negative effects on cells that were treated with PEVs, compared with other concentrations. For elemental analysis comparisons between PEVs and DEVs treatments, we selected a concentration of 100 EVs/cell. As a positive control treatment, we used 100 nM Dexamethasone (Dex; Sigma-Aldrich, Kenilworth, NJ, USA), which promotes the mineralization process in cells [32]. Untreated cells cultured in DMEM were used as a control sample. After 7 days post treatment, cells were harvested by trypsinization, lysed in 70% HNO_3_ overnight, and then, diluted to 1% HNO_3_ for elemental analysis with SN-ICP-MS.

### 2.7. Proliferation and Morphology of Cells on Ti Surfaces Coated with EVs (SurfEV)

#### 2.7.1. Cell Culture on Ti Surfaces

MG63 cells were seeded at the density 2.5 × 10^4^ cells/well on Ti disc surfaces with immobilized DEVs or PEVs (SurfEV) or coated with Basal Medium isolates as a control. DMEM (Dulbecco’s Modified Eagle’s Medium, Sigma-Aldrich, St. Louis, MO, USA) containing 10% of fetal bovine serum (FBS, Bovogen, Keilor East, VIC, Australia) and 1% of antibiotics (Pen/Strep) (100 units penicillin and 0.1 mg/mL streptomycin, Gibco, Grand Island, NE, USA) was used as a culture medium. Cells seeded on the SurfEV were incubated for 24 h at 37 °C and 5% of CO_2_.

#### 2.7.2. The Analysis of Cell Proliferation on SurfEV

To evaluate the proliferation of osteoblasts (MG63 cells) on SurfEV, a Cell Counting Kit 8 (CCK-8) assay was used according to previously described methodology (see #2.5.3). The proliferation rate was calculated according to the mean value of viability obtained for the control group (Ti samples without treatment).

#### 2.7.3. The Analysis of Cell Adhesion and Morphology on SurfEV

The adhesion and morphology of the MG63 cells seeded on SurfEV were assessed using scanning electron microscopy (SEM). MG63 cells were seeded on SurfEV and maintained in incubator at 37 °C and 5% of CO_2_ for 48 h. Cells were fixed with 2.5% of glutaraldehyde in 1 × PBS for 1 h at room temperature. After fixation, cells were washed with 1 × PBS three times for 5 min each and then with deionized water for 15 min twice. After washing, cells were dehydrated with 30%, 50%, 70% ethanol for 5 min twice in each concentration and with 90%, 100% ethanol for 5 min three times in each concentration. Finally, the samples were dried using the critical point dry method.

### 2.8. Statistical Analysis

For wound closure, cell proliferation individual cell migration tests and elemental analysis (ICP-MS) the numerical data were subjected to Shapiro–Wilk normality test. Once normal distribution was assumed, the analyzed data were presented as means and 95% confidence intervals (α = 0.05). For microscopy analysis, the results were descriptive (qualitative analysis).

## 3. Results

### 3.1. Characterization of Size, Size Distribution, Concentration, and Total Nucleic Acid Content of EVs

**Figure 1:**
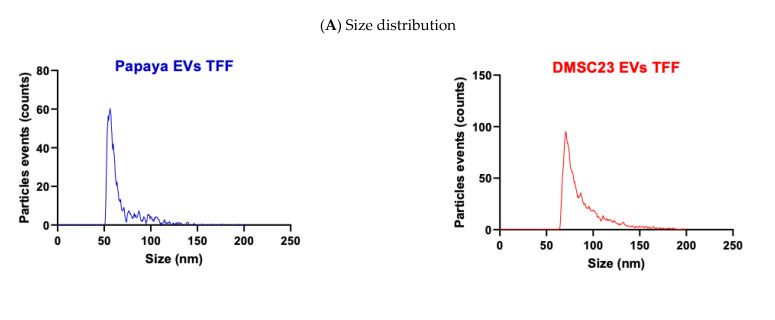

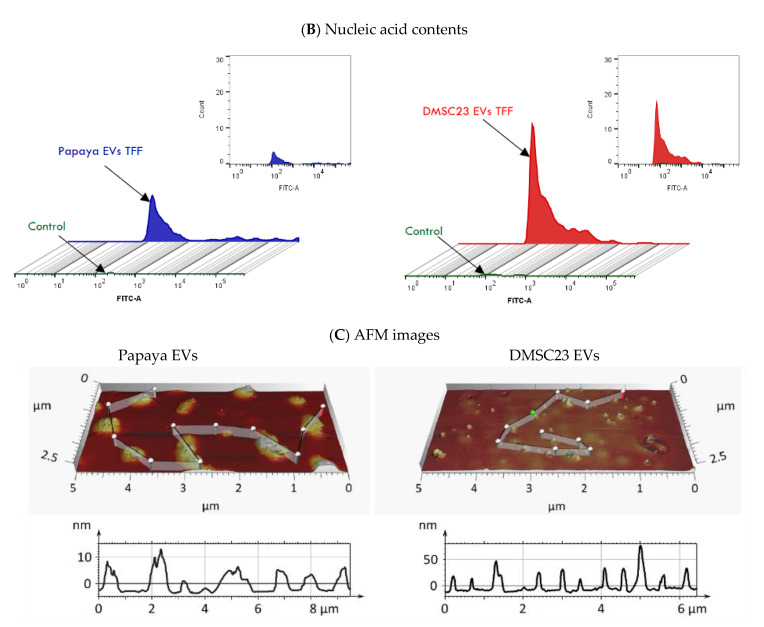
PEVs and DEVs characterization. (**A**) Size and size distribution–Nano FCM. (**B**) Composition of nucleic acid—Nano FCM. (**C**) Size and morphology of PEVs and DEVs—AFM.

The analysis using Flow NanoAnalyzer (NanoFCM Inc., Xiamen, China) showed that the average size of PEVs and DEVs was about 74 nm (Figure 1A). The size distribution of both PEVs and DEVs was homogenous (Figure 1A), and the EV size ranged between 50 and 140 nm and from 60 to 180 nm for PEVs and DEVs, respectively (Figure 1A). The concentration of nucleic acid in PEVs was 13.3%, while in DEVs, it was 39.4% (Figure 1B). AFM images showed that EVs had spherical morphology (Figure 1C). PEVs were partly agglomerated, and their height ranged from 5 to 12 nm, while DEVs were distributed individually, and their height ranged from 10 to 80 nm.

### 3.2. Investigation of the Presence of EVs on Ti Surfaces

**Figure 2:**
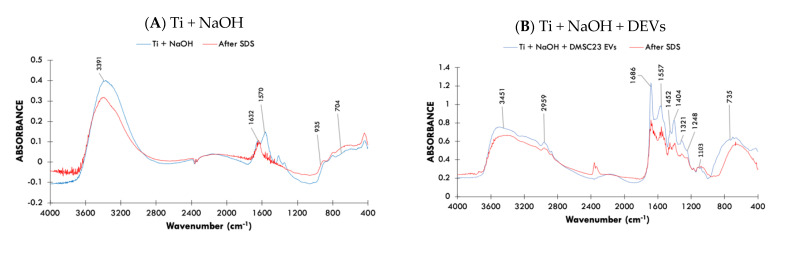

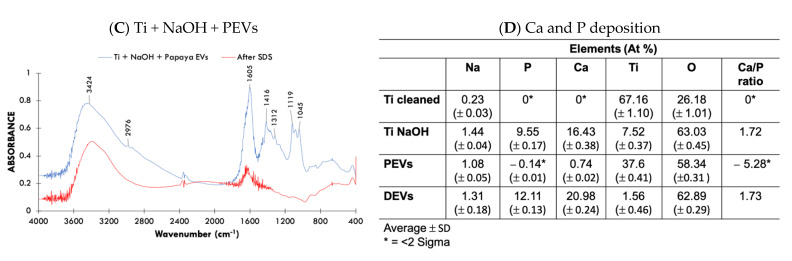
Physicochemical surface characterization. (**A**) FTIR spectrum of Ti + NaOH (**B**) FTIR spectrum of Ti + NaOH + DEVs (**C**) FTIR spectrum of Ti + NaOH + PEVs. (**D**) Ca and P deposition on Ti surface after 3 days in contact with SBF: (1) Cleaned; (2) NaOH; (3) NaOH + PEVs; (4) NaOH + DEVs. Results represent average of five areas ± SD values.

FTIR analysis showed that Ti surfaces treated with NaOH had two dominant peaks at 3391 cm^−1^ and 1632 cm^−1^. These two peaks are associated with O–H stretch vibration and bend vibrations, respectively, typically observed for NaOH treated Ti (Figure 2A) [33]. The water molecules are likely to be adsorbed to the surface during the treatment.

For samples coated with DEVs and PEVs, we observed additional peaks at 2959 cm^−1^ and 2976 cm^−1^ that corresponded to C–H stretch vibration. For the surface coated with DEVs, we observed two dominant peaks at 1686 cm^−1^, which corresponded to the amide I structure of proteins C=O [34], and 1557 cm^−1^, which corresponded to amide II arising N–H bending vibrations of the peptide groups [35]. The peak at 1452 cm^−1^ correlated with CH_3_ bend of the protein [17]. An additional peak at 1404 cm^−1^ confirmed the presence of a phosphatidylcholine bend [34], and the 1321 cm^−1^ peak corresponded to a C–N bend. The peak at 1248 cm^−1^ can be attributed to the RNA component, while the presence of 1103 cm^−1^ peak could be associated with phospholipids C–O stretch [36]. The peaks corresponding to proteins, nucleic acid (DNA, RNA), and lipids suggested the presence of DEVs on the Ti surface [17,36]. After the SDS washing, the peaks associated with DEVs remained present in the spectrum and were only partially diminished, which suggested robust immobilization of DEVs on the surface (Figure 2B).

For samples coated with PEVs, we observed the presence of a shoulder peak at 1645 cm^−1^ which corresponded to amide I [36] and one at 1416 cm^−1^ indicative of phosphatidylcholine bend [34]. The 1312 cm^−1^ peak suggested the presence of C–N bend. While peak at 1119 cm^−1^ was associated with phospholipid C–O stretch, and 1045 cm^−1^ peak with RNA [36]. Since these peaks are associated with organic compounds, this in turn suggested that PEVs were present on the Ti surface. However, when the surface was washed with SDS, peaks associated with PEVs on the surface were not present, which suggested removal or a substantial decrease of PEVs from the surface (Figure 2C); the spectrum appeared similar to the NaOH treated surface, which did not contain EVs immobilized on the surface (Figure 2A).

### 3.3. The Assessment Ca and P Deposition on Surfaces—Surface Bioactivity

The elemental analysis of the samples maintained for 3 days in SBF showed negligible amount of Ca/P deposited on untreated Ti samples (Ti cleaned). In contrast, both NaOH treated samples with and without DEVs induced the deposition of high amount of Ca/P on the surfaces (Figure 2D). The Ca/P ratio for both samples was greater than 1.67, meaning the apatite-formation ability on these surfaces [37]. For NaOH treated samples with PEVs, Ca/P was not detected on the surface (Figure 2D).

### 3.4. Effect of EVs Concentration on Cell Responses and Function

**Figure 3:**
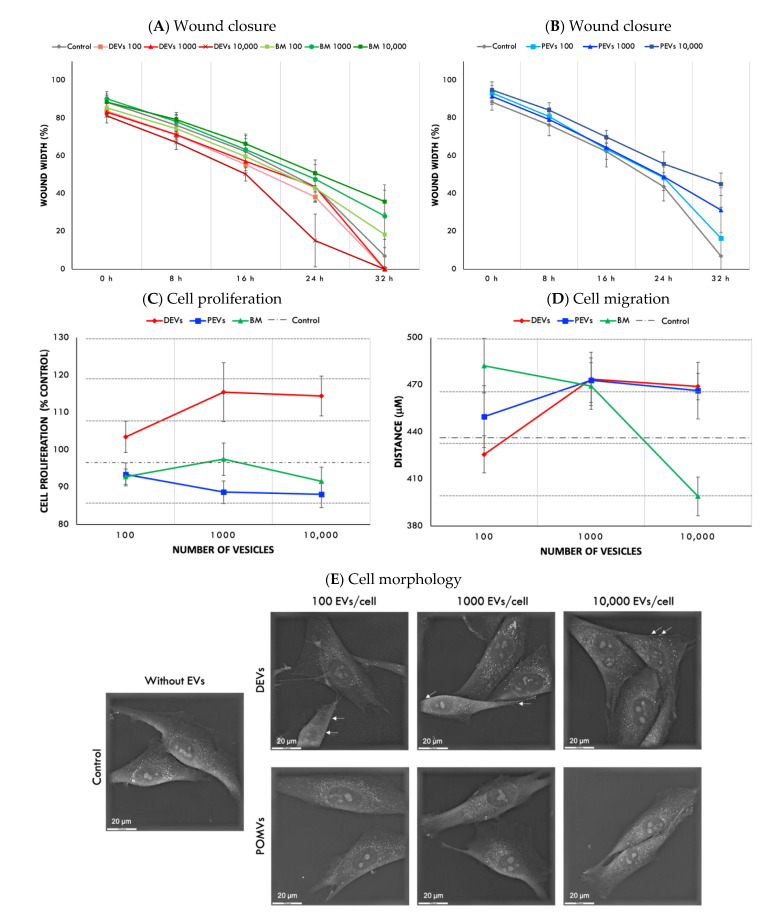
Effect of EVs on cell responses and function. (**A**) Wound closure of cells treated with DEVs and BM and (**B**) wound closure of cells treated with PEVs. Points represent means of two independent experiments carried out in quadruplicates and error bars show the confidence intervals (*α* = 0.05) of wound width (μm) at 0, 8, 16, 24, and 32 h, *n* = 8. (**C**) Osteoblast proliferation (% control), CCK-8, *n* = 8. Points represent means of two independent experiments carried out in quadruplicates and error bars show confidence intervals (*α* = 0.05). (**D**) Individual osteoblasts migration (total distance μm), Cell Tracking, *n* = 20. Points represent the means of two independent experiments, and error bars represent confidence intervals (*α* = 0.05). (**E**) Osteoblast morphology after 1 h in contact with EVs at different concentrations (NanoLive). White arrows indicate the presence of membrane exfoliate structures on the osteoblast surface (×60).

The wound healing assay showed DEVs at highest concentration (10,000 EVs per cell) and improved wound closure, particularly after 16 h (Figure 3A). The presence of a large standard deviation in this group occurred because some samples had their wound practically closed (Figure 3A). Cells treated with PEVs showed reverse correlation, lowest wound closure was observed in the group treated with PEVs at the highest concentration—10,000 EVs per cell (Figure 3B). None of the concentrations of BM isolates, used as DEV controls, increased the wound closure, as observed in Figure 3A at 32 h.

DEVs improved cell proliferation, however, no significant statistical difference was observed between 1000 vs. 10,000 DEVs (Figure 3C). Overall, the proliferation of cells treated with DEVs was ~15% higher than cells treated with BM (control experiment). Osteoblasts treated with PEVs showed a decrease of cell proliferation at all concentrations compared with control group (without EVs) (Figure 3C). In addition, cells treated with PEVs at 10,000 PEVs/cell concentration presented a substantial reduction on proliferation (~28%) when compared with cells treated with DEVs at the same concentration (10,000 DEVs/cell) (Figure 3C).

The analysis of individual cell migration showed that osteoblasts treated with DEVs and PEVs at 1000 and 10,000 EVs per cell concentrations travelled further compared with cells treated with DEVs and PEVs at the lowest concentration (100 EVs per cell). However, no statistical difference was observed when the concentration of DEVs and PEVs ranged from 1000 to 10,000 EVs per cell (Figure 3D). Decreased cell migration was observed when osteoblasts were treated with BM isolates particularly at the highest concentration (Figure 3D).

The analysis of cell structure and morphology using holotomography did not show noticeable differences between osteoblasts treated with PEV compared with control cells. On osteoblasts treated with DEVs, it was possible to identify some small, rounded membrane protruding structures on the osteoblasts’ surfaces (white arrows on Figure 3E). The protruding structures presented the same contrast as cell membrane and, potentially, are associated with the budding of secondary EVs from the cell membrane. In addition, we observed that, in the group treated at 100 DEVs per cell concentration, a large amount of these structures was found compared with groups treated with higher concentrations of DEVs (1000 and 10,000 EVs per cell).

### 3.5. Elemental Analysis of Cells Using Inductively Coupled Plasma Mass Spectroscopy—ICP-MS

**Figure 4:**
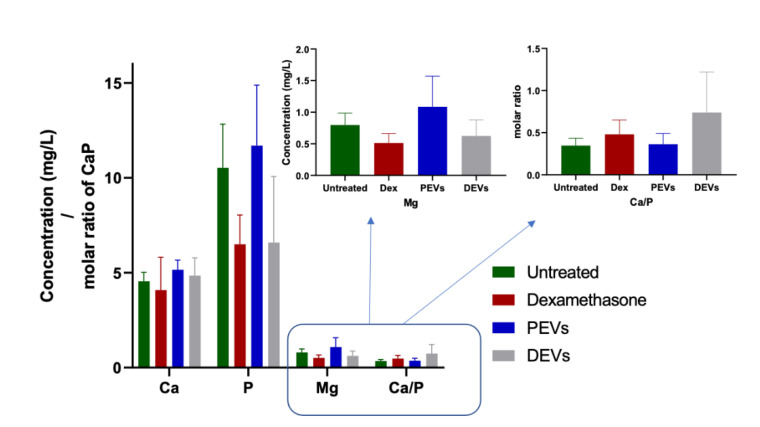
The quantification of intercellular Ca, P, Mg, and molar ratio of Ca/P. Results represent average of three independent experiments carried out in quadruplicate (SD values, *n* = 3).

The quantification of intercellular calcium showed that PEVs and DEVs increased the total calcium concentration in cells, respectively, by 13% and 7% when compared to untreated cells (control samples), while dexamethasone (Dex) treatment decreased the intercellular calcium concentration by ~10% (Figure 4). Further analysis showed that Dex and DEVs decreased the concentration of phosphorus, respectively, by 38% and 37% and magnesium by 36% and 22%. At the same time, PEVs increased the intercellular concentration of both phosphorus and magnesium by 11% and 36% when compared to untreated cells. The molar ratio of calcium and phosphate (Ca/P) for untreated cells was 0.34, and after the treatment with Dex and DEVs, the ratio increased, respectively, to 0.48 and 0.74 (Figure 4).

### 3.6. Proliferation and Morphology of Cells on Ti Surfaces Coated with EVs (SurfEV)

**Figure 5:**
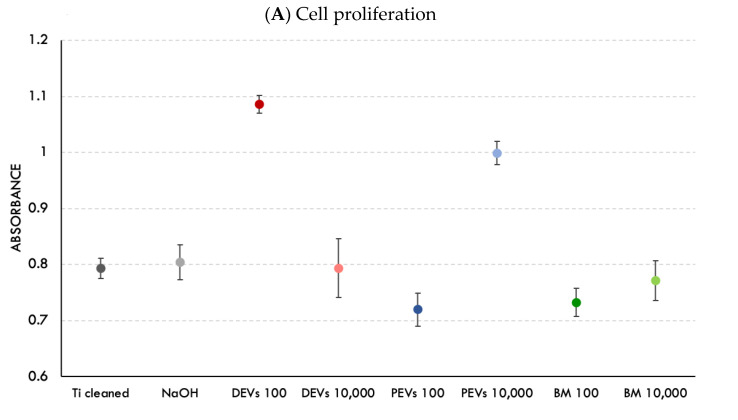

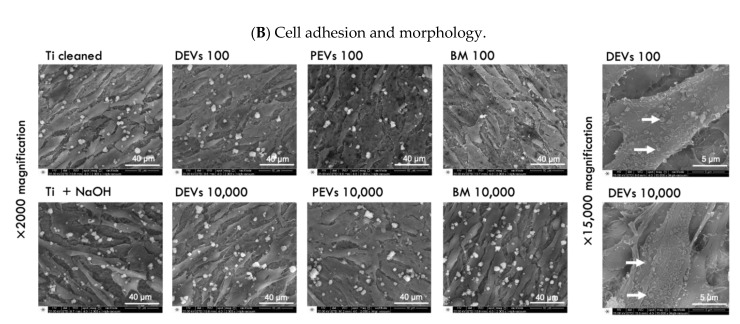
Proliferation and morphology of cells on SurfEV. (**A**) Osteoblast proliferation (% control), CCK-8, *n* = 8. Points are means of two independent experiments carried out in quadruplicate, and error bars represent confidence intervals (*α* = 0.05). (**B**) Osteoblast adhesion and morphology seeded on Ti surfaces coated with different EVs and the control groups (Ti cleaned and Ti treated with NaOH). SEM (×2000 and ×15,000 magnification). White arrows indicate the presence of membrane exfoliated structures on osteoblast cytoplasm.

Analysis of the ability of EV-coated surfaces (SurfEV) to promote cell proliferation showed that only surfaces coated with 100 DEVs and 10,000 PEVs per cell were able to increase cell growth. The other test and control samples, including 10,000 DEVs, 100 PEVs, 100 BM, 10,000 BM, Ti cleaned, and Ti treated with NaOH, showed similar cell proliferation (Figure 5A).

SEM images showed that cell density and adhesion for all samples was similar. However, cells cultured on DEV-coated surfaces had many characteristic protrusions on their cell membrane (white arrows, Figure 5B). Such protrusions are typical for cells secreting microparticles or other types of EVs, suggestive of active EV budding from the surface.

## 4. Discussion

Osteointegration is a dynamic process that allows implants to bond to/with bone and to fulfill their function [38]. A key element required to achieve osteointegration is the maintenance of physiological bone homeostasis at the implantation site. Bone homeostasis is controlled by cell-to-cell and cell-to-material interactions. If this process proceeds successfully it results in the stimulation of the cell growth and differentiation and leads to bone apposition on the implant surface [39]. To regulate cell–material interactions, implant surfaces are functionalized with various types of biomolecules that supposedly lead to desired integration of implants.

Since EVs mediate intracellular communication, it is conceivable that surfaces with immobilized EVs could stimulate cells that contact and surround the implant surface. In addition, direct transfer of proteins and miRNA from EVs to cells could regulate multiple cells signaling pathways (i.e., PI3K/Akt [16,17]) that are responsible for osteogenesis and osteoblastic differentiation of cells [40]. Furthermore, EVs derived from DMSCs reduce oxidative stress and inflammation, increase angiogenesis, and promote new bone formation in vivo under osteoporotic conditions [41,42,43]. Taken together, the immobilization of EVs on implant surfaces could potentially regulate and enhance the integration of implants in the body.

Here, we developed EV-functionalized surfaces using two types of EVs, which were derived from DMSC-23 cells (DEVs) and fermented papaya fluid (PEVs). Both types of EVs had similar size ~70 nm but were characterized with different amounts of nucleic acid; specifically, DEVs contained 39.4% and PEVs 13.4%. Differences in nucleic acid content and overall composition could have an impact of the biological function of EVs and their ability to promote implant integration in bone environment. To determine the effect of EV type and EV concentration on cell growth, we conducted a wound closure assay, measured cell proliferation, and determined the distance migrated by individual cells. Overall, we found that DEVs enhanced the closure of the wound, which increased with increasing DEV concentration (Figure 3A). Furthermore, our results showed that DEVs increased cell migration and cell growth (Figure 3A,C,D). In contrast, PEVs decreased the rate of the cell growth, and slowed down wound closure when compared with DEVs and control groups. These negative effects on cell growth were the most pronounced at the highest concentration of PEVs (10,000 PEVs/cell; Figure 3B,C). Interestingly, the analysis of individual cell migration showed that cells treated with PEVs had similar migration pattern (length and speed) to cells treated with DEVs, regardless of the concentration (Figure 3D). We attribute this result to the short 24-h period of analysis of the migration test. Overall, these effects of PEVs on cell growth and migration can be attributed to the origin of EVs and their composition [44].

A critical element of osteointegration is a synthesis of mineral phase by cells and the formation of calcium phosphates, i.e., hydroxyapatite–bone like structures. To demonstrate the ability of EVs to stimulate the formation of intracellular calcium phosphates, osteoblasts were treated with DEVs and PEVs, and the concentration of Ca and P was determined after 7 days. Both EV types increased intracellular concentration of Ca, P, and Mg compared to untreated cells. However, only cells treated with DEVs showed higher Ca/P ratio than untreated cells (negative control group). This suggests that DEVs can stimulate the formation of calcium phosphates and, potentially, could accelerate the mineralization process. Despite the increase of Ca/P ratio for DEV treated cells, we found that the ratio for all analyzed cells, including Dex treated cells (positive control), was under 1.2 (Figure 4). This result indicated that intercellular crystalline calcium phosphate was not fully developed, but its formation was stimulated by DEVs [45,46,47].

Detailed analysis of the Ca/P ratio showed that control cells and those treated with PEVs and Dex had a ratio of ~0.5, which corresponded with monocalcium phosphate. The Ca/P ratio of cells treated with DEVs was ~1.0, which corresponded with dicalcium phosphate dihydrate [47]. While previous studies indicated that Dex increases intracellular Ca/P [48], it was not observed in our experiments. This discrepancy might be due to the higher sensitivity of the methods we used to analyze the concentration of elements in our experiments.

Our analysis also showed differences in the intracellular Mg concentration depending on the treatment. Mg concentration is typically increased in the early stage of mineralization and gradually decreases and reaches a stable concentration in the late mineralization phase [49]. Therefore, decreased Mg concentration and increased Ca/P ratio for cells treated with DEVs suggested that DEVs stimulated the nucleation and mineralization process. This phenomenon was not observed for Dex treated cells, for which the Mg concentration decreased. Interestingly, in cells treated with PEVs, the Mg concentration increased; however, the Ca/P ratio was ~0.5. This result suggested that the nucleation and formation of Ca/P granules was at their early stages.

For EVs to be maximally effective in orthopedic implants, it is necessary to immobilize them on the implant surface. To achieve this, we developed a two-pronged approach, where surfaces were first chemically modified using alkaline treatment [26], then plasma activated [30], and finally, functionalized with EVs. In this way, the surface topography and chemistry were modified to facilitate physical entrapment of EVs and their chemical immobilization. The activation of the surfaces with plasma was applied to enhance chemical immobilization. Plasma treatment activates the surface by forming free radicals (unpaired electrons) on the surface that interact with EVs (e.g., surface proteins) and, potentially, covalently bind them. Chemical analysis using FTIR confirmed that DEVs were successfully immobilized on the surfaces. In contrast, FTIR analysis showed that PEVs were not covalently attached and, after washing with detergent, EVs were not detected on the surface. Since DEVs and PEVs are derived from different cells, it is likely that their surface composition is different, thus, they show different affinity to our surfaces. DEVs contain protein surface markers and may contain proteins and other biomolecules directly on their surface. These biomolecules can interact with free radicals on the implant surface and form a stable bond.

While the effects of both types of EVs on cell growth in solution were confirmed, it was necessary to determine whether EVs immobilized on the surface (SurfEV) remained active and induced the same effects. We found that cell proliferation was enhanced only at specific EV concentrations (Figure 5A). DEVs at concentrations of 100 EVs/cell and PEVs at 10,000 EVs/cell considerably increased cell proliferation. Although we observed the increase in cell proliferation for the group treated with PEVs at highest concentration (Figure 5A), the overall assessment of cell responses showed that PEVs led to the decreased rate of wound closure and did not improve mineralization.

The analysis of cell morphology showed the desired adhesion of osteoblasts on all tested surfaces. However, for surfaces functionalized with DEVs, we found the presence of many protruding structures on the cell membrane, which potentially were associated with budding of secondary EVs (Figure 5B). It is likely that DEVs promoted the secretion of EVs, which subsequently, could have additional effect on surrounding cells. In summary, we showed that DEV functionalized surfaces enhance cell proliferation and activate osteoblast-secreted EVs, while PEV functionalized surfaces only increased cell proliferation at higher concentrations (10,000 PEVs/cell).

After confirming the ability of the surface to immobilize EVs, we measured the formation of hydroxyapatite on surfaces when immersed in simulated body fluid (also known as the bioactivity test). This test measures the ability of an implant to bond with bone [29,37]. We showed that the concentration of Ca and P significantly increased on DEV-functionalized surfaces when compared with control samples. PEVs-functionalized surfaces did not promote detectable deposition of Ca and P on the surface. In addition, Ca/P ratio for NaOH treated and DEVs coated surfaces was >1.67, suggesting the formation of hydroxyapatite on the surface [35], which is considered a predictor of bioactivity and the implant’s ability to integrate with bone [50].

Even though data from this in vitro study cannot be fully extrapolated to clinical conditions, it was found that immobilizing specific EVs can regulate the cell responses (proliferation, migration, mineralization) that are likely to promote and enhance osteointegration process. The functionalization of the implant surfaces with EVs can be an effective strategy to aid implant integration in the body via active stimulation of cells that surround the implant. While the SurfEV showed promise to aid implant integration within the body environment, further pre-clinical experiments are required to demonstrate their effectiveness. However, as demonstrated through our work, the composition of EVs can be modulated; hence, it is possible to fabricate EV coatings that are ‘personalized’ for different implants or even patients. For example, dental implants should stimulate osteointegration, while gynecological or urological implants should promote integration with soft tissue. This means that we can select EVs that have composition tailored for each of these applications.

## 5. Conclusions

Based on the results, we conclude that EVs derived from placenta-derived stem cells—DEVs—increased osteoblast proliferation, enhanced wound closure, and increased individual cell migration in terms of both distance and speed of the cell movements. Based on the elemental analysis of cells treated with both classes of EVs, we found that DEVs accelerated the nucleation of calcium phosphates, thus, promoting the mineralization process. In contrast, PEVs had no effect on the formation of the mineral phase when compared with control, untreated cells. However, to enable integration of implants, it is essential to immobilize EVs on the surface. We showed that only DEVs formed stable chemical bond with the nanostructure and plasma-activated surfaces. EV-functionalized surfaces were called SurfEV. We showed that immobilized EV maintained their activity and enhanced cell growth and stimulated apposition of calcium phosphates directly on the surface. These results suggest that SurfEV improved bioactivity of the titanium, which is a prerequisite for functional integration of orthopedic implants in the body.

## Figures and Tables

**Table 1:** Groups for FTIR analysis.

Sample	Treatment
1	Ti cleaned
2	Ti + NaOH + plasma
3	Ti + NaOH + plasma + DEVs
4	Ti + NaOH + plasma + PEVs

Defined experimental groups.

## Data Availability

Data presented in this article is available at request from the corresponding author.
